# Quantification of cephalocaudal progression of jaundice in preterm infants

**DOI:** 10.1038/s41390-022-02396-y

**Published:** 2022-11-28

**Authors:** Alida J. Dam-Vervloet, Foky-Anna de Boer, Ingrid M. Nijholt, Lieke Poot, Nienke Bosschaart, Henrica L. M. van Straaten

**Affiliations:** 1 Medical Physics Department, Isala Hospital, Zwolle, The Netherlands; 2 Biomedical Photonic Imaging group, Technical Medical Centre, University of Twente, Enschede, The Netherlands; 3 Neonatology Department, Isala Hospital, Zwolle, The Netherlands; 4 Innovation & Science Department, Isala Hospital, Zwolle, The Netherlands; 5 Radiology Department, Isala Hospital, Zwolle, The Netherlands

## Abstract

**Background:**

The cephalocaudal progression (CCP) of neonatal jaundice is a well-known phenomenon, but quantitative information on CCP in preterm infants is absent. In this study, CCP was quantified in preterm infants as a function of postnatal age and body location.

**Methods:**

5.693 transcutaneous bilirubin (TcB) measurements were performed in 101 preterm infants from birth until postnatal day seven at five body locations (forehead, sternum, hipbone, tibia, ankle). Multi-level linear regression analysis was performed to evaluate the CCP as a function of body location and postnatal age. TcB measurements at all body locations and postnatal days were compared to total serum bilirubin (TSB) levels (*N* = 1.113).

**Results:**

The overall average change in ratio of TcB compared to forehead was for sternum +0.04 [95% CI −0.02;0.09]; hipbone +0.05 [0.00;0.01]; tibia −0.33 [−0.38;−0.27] and ankle −0.62 [−0.68;−0.57]. No effect modification of CCP by sex, gestational age, birthweight, phototherapy, and TSB was found. The TcB maximally underestimated the TSB at the ankle −79.5 µmol [−0.1;159.2].

**Conclusions:**

CCP is present in preterm infants and is relatively stable over time. Since TcB measurements on the tibia and ankle underestimate TSB significantly, we advise to use only measurement locations cephalic from the tibia; i.e., hipbone, sternum, and forehead.

**Impact:**

- Cephalocaudal progression (CCP) of jaundice in preterm infants, assessed by transcutaneous bilirubin (TcB) measurements, is substantial and rather stable over postnatal day 0 to 7.
- To the best of our knowledge, this study is the first to investigate CCP of jaundice in preterm infants as a function of postnatal age in preterm infants.
- Our results demonstrate that TcB measurements at the tibia and ankle differ from the TSB beyond the clinically used TcB safety margins. We advise to perform TcB measurements only at locations cephalic from the tibia; i.e., hipbone, forehead, and sternum.

## Introduction

Jaundice in newborn infants is a common clinical condition. It affects up to 80% of preterm and 60% of the term newborn infants.^1^ Neonatal jaundice can potentially progress to severe hyperbilirubinemia, which may result in kernicterus spectrum disorders (KSDs) or bilirubin-induced neurologic dysfunction (BIND), causing irreversible brain damage.^2^ Timely treatment of jaundiced newborn infants with phototherapy can prevent severe hyperbilirubinemia and its neurological sequelae as well as the need for invasive treatment (e.g., blood exchange transfusion).^3^ Therefore, screening of newborn infants at risk for hyperbilirubinemia is important and advised in guidelines.^4,5^ Transcutaneous bilirubinometry (TcB) is an effective non-invasive method for screening hyperbilirubinemia and it can reduce the number of invasive total serum bilirubin (TSB) determinations, which is currently considered the golden standard.^6–10^

The yellow skin discoloration associated with neonatal jaundice is often first observed at the face and only later in the extremities. This phenomenon is known as the cephalocaudal progression (CCP) of jaundice. The physiological causes and spatiotemporal dependency of CCP are still not fully understood.^11,12^ More insight into this phenomenon can improve our in-depth understanding of the progression and diagnosis of hyperbilirubinemia. From a more practical point of view, it is important to understand the influence of CCP on the correlation between the TcB and TSB. Current transcutaneous bilirubin (TcB) meters are designed for forehead and/or sternum measurements. However, measurements on other body locations are gaining popularity, e.g., on the interscapular site to avoid the influence of ambient light^13^ and at the hipbone underneath the diaper to reduce the influence of photototherapy.^14^ CCP potentially influences the reliability of the TcB at these non-standard body locations.

Previous studies assessed CCP based on TcB measurements in jaundiced newborn infants. Hegyi found a ratio of TcB measurements of the sole compared to the forehead of 0.58 (SD 0.07) for clinically healthy newborn infants (unknown gestational age (GA));^15^ Knudson demonstrated a significantly higher TcB at the forehead compared to the sole (ratio unknown) for newborn infants with a median GA of 40 weeks;^16^ Purcell reported a significantly higher TcB value on the head compared to the sole (ratio unknown) for newborn infants with an average GA of 38.9 weeks;^17^ and Kamphuis found a foot/ forehead ratio of 0.44 (SD 0.15) for newborn infants with a median GA of 37.1 weeks.^18^

Besides term infants, preterm infants are also often subjected to TcB measurements. To the best of our knowledge, the CCP of jaundice has not yet been investigated in preterm infants. As CCP may also influence the reliability of TcB measurements in this patient group, our aim was to evaluate the presence of CCP in preterm infants and to quantify it as a function of time (postnatal day 0–7) and body location.

## Methods and materials

### Study population

Preterm infants (≥28 weeks GA), born between December 2017–September 2019 admitted to the NICU at Isala Women and Children’s hospital (Zwolle, the Netherlands) were included after informed consent was obtained from the parents. Hypothermia treatment was an exclusion criterium, since it may influence bilirubin metabolism and thereby the transcutaneous measurement outcome.^19^

This study was approved by the Medical Ethical Committee of Isala Hospital in Zwolle, the Netherlands (number 170317).

### TcB meter

Transcutaneous measurements were performed using the transcutaneous meter type JM-105 (serial numbers: B3601027 and B3601086, Draeger Medical, Lubeck, German), which is widely used in pediatric and neonatal departments across the Netherlands. The accuracy of the TcB measurements specified by the manufacturer is 25.5 μmol/L (>35 weeks GA) and 27.4 μmol/L (>24 weeks GA).^20^ Care was taken to use the same TcB meter per measurement series, in order to avoid the potential influence of low inter-device reproducibility.^21^

### TcB measurements

From birth (postnatal day 0) until postnatal day seven, TcB measurement series at different body locations were ideally performed at least three times a day for each patient. Owing to practical reasons, it was not always possible to perform three measurements every day. The TcB measurement series were performed by the nurse or attending physician during planned care (e.g., during physical examination or routine nursing care), preventing any avoidable disturbance. All nurses and physicians on the NICU were trained in the proper use of the TcB meter. To evaluate the presence of CCP of jaundice, every measurement series included five different body locations (forehead, sternum, hipbone, tibia, ankle), representing each dermal zone described by Kramer.^22^ Measurements were performed on uncovered skin. Hipbone (spina iliaca anterior superior), tibia (tibialis medialis, approximate 1 cm below tuberositas tibia, representing the same location used for intra-osseous acces) and ankle (malleolus medialis) measurements were performed at the best reachable side of the body (left or right), depending on patient orientation. Each TcB measurement was the average of three repeated measurements per location, as suggested in the manufacturer’s instructions.^20^ This means that 15 measurements (5 locations x 3 repeated measurements) were performed per patient, per TcB measurement series. Our experienced staff could finish these 15 measurements within a few minutes.

### Cephalocaudal progression

Cephalocaudal progression (CCP(*x*, *t*)) was quantified as the ratio of the TcB_*x,t*_ and the corresponding TcB_forehead,*t*_ minus the reference ratio of the forehead (TcB_forehead*,t *_*/* TcB_forehead*,t*_ = 1):

$${{{{{\mathrm{CCP}}}}}}\left( {x,t} \right) = \left({{{{{{{\mathrm{TcB}}}}}}}_{x,t}/{{{{{\mathrm{TcB}}}}}}_{{{{{{\mathrm{forehead}}}}}},t}} \right)-1$$

The subscript *x* denotes the measurement locations sternum, hipbone, tibia and ankle and *t* denotes the postnatal age. We chose to express CCP as a ratio difference rather than a difference of absolute values, because of the large dispersion in absolute TcB values between patients. In this way, we could determine an average relative change, which can be applied to the full range of TcB values. A negative (–) CCP implies that *TcB*_*x,t*_ is lower than *TcB*_forehead,*t*_ and vice versa for a positive (+) progression. The CCP was determined for all postnatal ages combined and per postnatal day.

Since multiple measurement series were performed over time, a multi-level linear regression analysis (STATA version 15, StataCorp LLC, Texas) was applied to assess CCP with its 95% confidence interval (CI). For this analysis, measurements were clustered per patient. To check for effect modification of CCP, interaction terms of the following variables were added to the analyses; sex, GA, birthweight, phototherapy and TSB. TSB was analyzed both as a continuous and dichotomous measure, for which the threshold <170 μmol/L was used as cut-off value. A *p*-value of <0.05 was considered statistically significant.

### Correlation of TcB measurements

Three repeated measurements per location were performed. Repeated measurement correlation was applied to determine how well the individual TcB measurements correlated with each other (*R*_rep_) using mrcorrShiny (Version 2, University of Texas).^23,24^ For all repeated measures analysis the measurements were clustered per patient.

### Agreement of TcB measurements and TSB measurements

Serum bilirubin levels in arterial or capillary blood samples were determined by the ABL-90 Flex Plus bloodgas analyzer (Radiometer, Brønshøj Denmark). TcB measurements were paired in time as much as possible with routine invasive blood sampling. Measurements of TSB and TcB within a time frame of 1 h were labeled as “paired” (Table 3). Since Isala hospital facilitates comprehensive point-of-care-testing (POCT), TSB levels were available from each bloodgas-analysis, resulting in multiple paired TcB-TSB measurements per patient.

The agreement between TSB and TcB levels was calculated (TSB*–*TcB_*x,t*_) and depicted in Bland-Altman plots, adjusted for repeated measures for all body locations, using MedCalc (MedCalc Software Version 20.026, Ostend, Belgium).^25^ Repeated measurement correlation was applied to determine how well the TcB measurements correlated with the paired TSB measurements (*R*_TcB,TSB_) using mrcorrShiny (Version 2, University of Texas).^23,24^ For all repeated measures analysis the measurements were clustered per patient.

## Results

From 101 newborns, 5.693 TcB and 1.113 TSB measurements were included for data analysis (Tables 1 and 2).

**Table 1:** Patient characteristics.

Number of included patients	101
Prematurity^26^	
Very preterm (28 to 32 weeks of gestation)	91 (90%)
Moderate to late preterm (32 to 37 weeks of gestation)	10 (10%)
Gestational age, weeks (average ± SD [range])	30^+3^ ± 1^+5^ [28–35]
Sex	
Male	60 (59%)
Female	41 (41%)
Ethnicity	
Caucasian	66 (66%)
Other (1x Asian, 1x Latin American, 1x African, 1x Turkish, 2x Other)	6 (6%)
Unknown^a^	29 (29%)
Birthweight, gram (average ± SD [range])	1518 ± 448 [675–3280]
Number of TcB measurements	5.693
Number of TSB measurements	1.113

^a^The majority of the unreported ethnicity was expectedly Caucasian.

**Table 2:** Number of TcB ratios per measurement over postnatal day (0–7) and body location and averaged ratio TcB_*x*,*t*_/TcB_forehead,*t*_ over all postnatal ages.

	Number (TcB_*x,t*_/TcB_forehead,*t*_) measurements per postnatal day								Total number of (TcB_*x*,*t*_/TcB_forehead,*t*_) measurements over all postnatal ages combined	Average ratio (TcB_*x*,*t*_/TcB_forehead,*t*_) over all postnatal ages combined (SD)
	0	1	2	3	4	5	6	7	0–7	0–7
TcB_forehead_/TcB_forehead_	110	219	107	109	196	168	137	105	1.151	1.00 (± 0)
TcB_sternum_/TcB_forehead_	110	216	106	107	191	165	136	102	1.133	1.04 (±0.52)
TcB_hipbone_/TcB_forehead_	110	218	107	107	191	166	136	104	1.139	1.05 (±1.29)
TcB_tibia_/TcB_forehead_	110	217	106	108	192	164	136	105	1.138	0.67 (±0.56)
TcB_ankle_/TcB_forehead_	110	213	106	108	192	163	135	105	1.132	0.38 (±0.40)
Total number of TcB_x_/TcB_forehead_	550	1083	532	539	962	826	680	521	5.693	

### Cephalocaudal progression of the TcB measurements

**Fig. 1:**
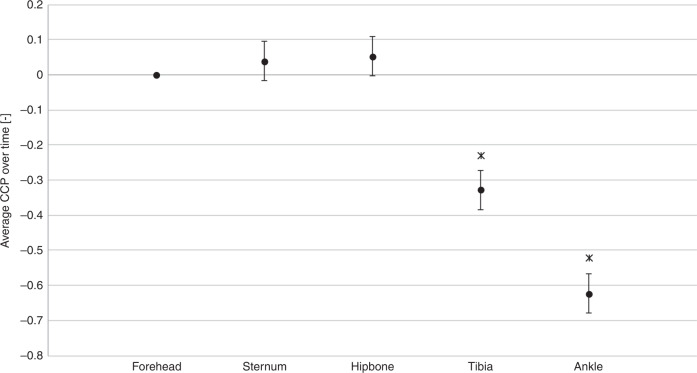
Cephalocaudal progression of jaundice. Average CCP(*x*,*t*) over all postnatal days combined (*t*) for all locations (*x*), with corresponding 95% confidence intervals (error bars). Locations where average CCP(*x*,*t*) was significantly different from 0 are marked with a ж. CCP of sternum and hipbone were not significantly different from 0.

The averaged CCP(*x*,*t*) over all postnatal ages combined was not significantly different from 0 (*p* = 0.175 and *p* = 0.067, respectively) for the sternum (+0.04 [95% CI −0.02; 0.09]) and the hipbone (+0.05 [95% CI 0.00; 0.01]) (Fig. 1). The CCP over all postnatal ages combined was significantly lower than 0 for the tibia (−0.33 [95% CI −0.38; −0.27]) and the ankle (−0.62 [95% CI −0.68; −0.57]).

**Fig. 2:**
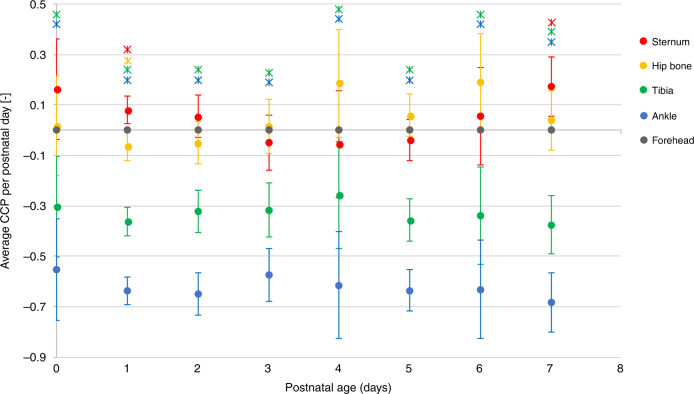
Cephalocaudal progression of jaundice as a function of postnatal age. Average CCP(*x*, *t*) per location (*x*) for all postnatal days (*t*), with corresponding 95% confidence intervals (error bars). Locations where average CCP(*x*, *t*) significantly different from 0 are marked with a ж with corresponding measurement location color.

The CCP(*x*,*t*) at the sternum (ranging from −0.06 [95% CI −0.27; 0.16] to 0.17 [95% CI 0.06; 0.29]) and the hipbone (ranging from −0.06 [95% CI –0.12; 0.01] to 0.19 [95% CI −0.03; 0.40]) was not significantly different from 0 on all individual postnatal days, except on postnatal days 1 and 7 for the sternum and day 1 for the hipbone (Fig. 2). The CCP(*x*,*t*) was significantly lower than 0 for the tibia (ranging from −0.37 [95% CI −0.49; −0.26] to −0.26 [95% CI −0.47; −0.04]) and the ankle (ranging from −0.68 [95% CI −0.80; −0.56] to −0.55 [95% CI −0.75; −0.35]) on all postnatal days.

None of the variables sex, gestational age, birthweight, phototherapy and TSB were observed to be an effect modificator of the overall change in ratio between the various locations.

### Correlation of TcB measurements

The repeated measurement correlation between the individual TcB measurements at the forehead, sternum, hipbone and tibia was similar and strong (*R*_rep_ = 0.88, 0.87, 0.84, and 0.87, respectively), whereas the repeated measurement correlation between the individual TcB measurements at the ankle was substantially weaker (*R*_rep_ = 0.67) (Table 3).

**Table 3:** Paired TcB-TSB measurements.

Body location	Number of paired TcB-TSB measurements	Average TcB (µmol/L)		Average TSB (µmol/L)		Repeated measurement correlation TcB (*R*_rep_)	Correlation between TcB and TSB (*R*_TcB,TSB)_)
		Average	SD	Average	SD		
Forehead	648	126	58	119	52	0.88	0.84
Sternum	637	127	67			0.87	0.74
Hipbone	645	119	58			0.84	0.80
Tibia	644	81	49			0.87	0.81
Ankle	641	46	35			0.67	0.56

TcB and TSB levels and corresponding correlations (*R*_TcB,TSB_) for the five cephalocaudal body locations. The repeated measurement correlation (*R*_rep_) is listed in the fifth column.

### Agreement of TcB measurements and TSB measurements

**Fig. 3:**
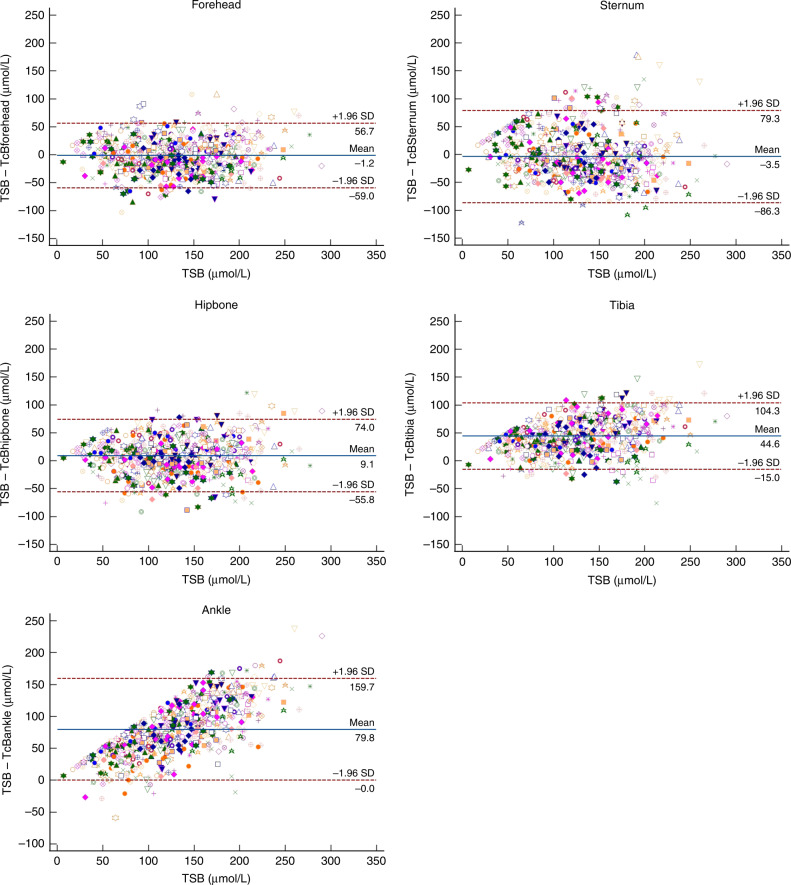
Bland–Altman plots for all paired TcB-TSB measurements at the five cephalocaudal body locations. The blue horizontal lines represent the average differences between TcB and TSB: −1.2, −3.5, 9.1, 44.6, 79.8 μmol/L for the forehead, sternum, hipbone, tibia and ankle respectively. The red lines represent the upper en lower limits of agreement. Bland–Altman was corrected for repeated measures, where measurements were clustered per patient. 17.1 μmol/L = 1 mg/dL bilirubin.

Approximately 640 paired TcB-TSB measurements were included per location (Table 3). Figure 3 presents Bland–Altman plots that compare the TcB to the TSB for all paired measurements per body location. The TSB was overestimated by TcB levels at the forehead (−1.2 µmol [lower limit of agreement −59.0; upper limit of agreement 56.7]) and sternum (−3.5 µmol [−86.3; 79.3]). The TSB was underestimated by TcB levels at the hipbone (9.1 µmol [−55.8; 79.3]), tibia (44.6 µmol [−15.0; 104.3]), and ankle (79.8 µmol [−0.0; 159.7]) (Fig. 3). The correlation between the TSB and TcB at the forehead, sternum, hipbone and tibia was similar (*R*_TcB,TSB_ = 0.84, 0.74, 0.80 and 0.81, respectively), whereas the correlation between the TSB and TcB at the ankle was substantially lower (*R*_TcB,TSB_ = 0.56) (Table 3).

## Discussion

The main purpose of this study was to evaluate the cephalocaudal progression of jaundice in preterm infants as a function of time (postnatal age day 0–7) and body location. Hereto, we evaluated TcB measurements on five cephalocaudal body locations (forehead, sternum, hipbone, tibia and ankle). We showed that CCP was present and stable over time. The CCP(*x*,*t*) over all postnatal ages combined was 0.04 for the sternum, 0.05 for the hipbone, and significantly lower for the tibia (−0.33) and the ankle (−0.62). Sex, gestational age, birthweight, phototherapy and TSB were no effect modificators of the CCP ratio changes between the locations. Furthermore, TcB measurements at the forehead, sternum and hipbone were in agreement with the TSB within the reported accuracy by the manufacturer (27.4 μmol/L).^20^ On body locations caudal from the hipbone, the TcB underestimated the TSB.

Our results on the CCP of jaundice on preterm infants (average GA of 30^+3^ weeks) are in line with previous studies on term newborn infants.^15–18^ CCP ratios of TcB measurements on caudal sites were similar to the reference ratio at the forehead and decreased towards the caudal side of the body.^15–18^ For instance, our CCP ratio of the ankle compared to the forehead of 0.38 (SD 0.40) is comparable to Kamphuis 0.44 (SD 0.15).^18^ In accordance with previous studies, our study demonstrated a good correlation between TSB and TcB measurements on forehead, sternum and hipbone.^14,16^ In addition, we found a good correlation on the tibia.

A strength of our study is the large study population and the high number of TcB measurement series and TcB-TSB pairs. Owing to the high number of measurements, we were able to perform repeated measures analysis. In many validity and correlation analyses, there is often no correction applied for repeated measurements although repeated measurements were done. This may produce biases, specious results due to violation of independence and/or differed patterns between-participants versus within-participants.^23–25^ In our study, we performed multi-level linear regression analysis, Bland–Altman and correlation analyses between TSB and TcB levels with correction for repeated measures.

### Study limitations

Our results cannot be generalized to the entire preterm newborn population, because the majority of our study population was Caucasian and TcB measurements may vary with other skin types. Several studies showed that darker skin tones can lead to overestimated TcB values.^26–29^ However, while other studies confirmed the fact that TcB tends to overestimate TSB for darker skin tones, they also showed the reliability of the TcB to assess TSB regardless of skin color.^29–31^

In our recent work, we demonstrated that local skin anatomy can influence TcB readouts,^32^ which is an important aspect to take into account when evaluating the TcB at different body locations. Furthermore, Purcell et al. found that the measured TcB, skin temperature and capillary refill time show a similar cephalocaudal progression.^17^ Variations in skin maturity (optical scattering), bone depth and potentially skin temperature can influence the measured TcB significantly.^17,32^ For this study, skin maturity at the same postnatal day can be considered to be a constant factor in a patient over different body locations. Other studies confirm that this factor does not differ significantly between the evaluated body locations.^33^ Therefore, we assume that only bone depth and skin temperature can have a potential influence on our cephalocaudal TcB measurements. Since TcB measurements are underestimated beyond the accuracy specified by the manufacturer 27.4 μmol/L^20^ for bone depths <1.1 mm,^32^ we expect that this effect would be most prominent for the most premature infants in our study population. However, we did not observe any influence of gestational age on our results. To fully take into account the influence of local skin anatomy and skin temperature on cephalocaudal TcB measurements, follow up research may benefit from the use of spectroscopic high-resolution skin imaging and local skin temperature measurements. High-resolution skin imaging can be achieved with either high-resolution ultrasound^34^ optical coherence tomography.^35^ The latter combines high-resolution skin images with spatially confined bilirubin measurements, which may provide even further in-depth understanding into the relation between microcirculatory perfusion and the extravasation of bilirubin into the skin and local temperature measurements.^35^

### Clinical implications

This study provides insight into the CCP of jaundice in preterm infants as a function of postnatal age and body location. With a CCP of the ankle up to −0.70 and a mean underestimation of the TSB at the ankle of 80 μmol/L, our results demonstrate that body location affects the measured TcB. Furthermore, our results demonstrate that the clinically accepted safety margin (50 μmol/L below the phototherapy threshold) can be exceeded on more caudal body locations.^4,6^ Therefore, healthcare providers should be aware that the decision to do an additional TSB determination can be influenced by the TcB measurement location. Based on our observations, we advise to only use measurement locations cephalic from the tibia; i.e., hipbone, sternum and forehead.

## Conclusion

In this study, we demonstrated the presence of cephalocaudal progression of jaundice in preterm infants during the first week of life. The TcB at the tibia and ankle differed significantly from the TcB at the forehead throughout the evaluated period. The measured CCP ratios remained relatively stable over time. No effect modification by sex, gestational age, birthweight, phototherapy and TSB was found. The deviation of the TcB from the TSB exceeded the clinically accepted safety margins for the ankle and tibia with an average underestimation of 45 and 80 μmol/L, respectively. Based on the results of this study, we advise to use only TcB measurement locations cephalic from the tibia; i.e., hipbone, sternum, and forehead.

## Data Availability

The data that support the findings of this study are available from the corresponding author, Dam-Vervloet, upon reasonable request.
